# Intraocular Pressure Response to Short-Term Extreme Normobaric Hypoxia Exposure

**DOI:** 10.3389/fendo.2018.00785

**Published:** 2019-01-07

**Authors:** Eliška Najmanová, František Pluháček, Michal Botek, Jakub Krejčí, Jana Jarošová

**Affiliations:** ^1^Department of Optics, Faculty of Science, Palacký University Olomouc, Olomouc, Czechia; ^2^Department of Natural Sciences in Kinanthropology, Faculty of Physical Culture, Palacký University Olomouc, Olomouc, Czechia

**Keywords:** intraocular pressure, hypoxia, normobaric hypoxia, glaucoma, oxygen saturation

## Abstract

**Purpose:** The purpose of the study was to determine the intraocular pressure response to normobaric hypoxia and the consequent recovery under additional well-controlled ambient conditions. Second, the study attempted to determine if the intraocular pressure changes were dependent on its baseline, initial heart rate, sex and arterial oxygen saturation.

**Methods:** Thirty-eight visually healthy volunteers (23 women and 15 men) of an average age 25.2 ± 3.8 years from 49 recruited participants met the inclusion criteria and performed the complete test. Initial intraocular pressure (baseline), heart rate, and arterial oxygen saturation were measured after 7 min of rest under normal ambient conditions at an altitude 250 m above sea level. Each subject then underwent a 10 min normobaric hypoxic exposure and a subsequent 7 min recovery under normoxic conditions. Within hypoxic period, subjects were challenged to breathe hypoxic gas mixture with fraction of inspired oxygen of 9.6% (~6.200 m above sea level). Intraocular pressure and arterial oxygen saturation were re-measured at 4 and 10 min during the hypoxia and at 7 min after hypoxia termination.

**Results:** Intraocular pressure increased in 1.2 mmHg ± 1.9 mmHg and 0.9 mmHg ± 2.3 mmHg at 4 and 10 min during the hypoxic period and returned approximately to the baseline at 7 min of recovery. The influence of sex was not statistically significant. The arterial oxygen saturation decreased in 14.9 ± 4.2% at min 4 and 18.4 ± 5.8% at min 10 during hypoxia and returned to the resting value at 7 min of recovery. The decrease was slightly higher in the case of women if compared with men. The hypoxia induced changes in intraocular pressure were significantly correlated with the arterial oxygen saturation changes, whereas the relationship with intraocular pressure baseline and initial heart rate were insignificant.

**Conclusion:** There was a significant increase in intraocular pressure as a response to short-term normobaric hypoxia, which returned to the baseline in 7 min after hypoxia. The increase was dependent on the induced oxygen desaturation.

## Introduction

Active vacations at high altitudes such as skiing, heli-skiing, hiking, and mountain climbing have become increasingly popular for people all around the world. Due, however, to rapid modern transportation patterns (lifts, cars, helicopters, airplanes), it is currently easy to passively reach an altitude over 2,500 m high, and people without appropriate acclimatization to hypobaric hypoxia may start experiencing symptoms related to acute mountain sickness for instance with headache, fatigue, nausea, or gastrointestinal issues, and in severe phases also by pulmonary oedema and/or high-altitude cerebral oedema (1). Moreover, various altitude or hypoxic activities are included to the training strategies of elite athletes (2). Such activities should have consequences in their health status including ocular health.

It was demonstrated that the altitude changes influence the intraocular pressure (IOP) (3–6), however, the underlying mechanism is unclear, and therefore needs an explanation. The IOP is one of the primary observed and important parameters connected with the second leading cause of blindness in the world-glaucoma (7). Normal IOP values, which maintain the integrity of the eye without optic nerve damage, are in a range between 10 and 20 mmHg (8). Higher IOP or its rapid changes are considered a risk factor for development of glaucoma changes (7). The people with glaucoma also show greater short-time IOP fluctuations (9). The IOP level is related to the dynamic parameters of the aqueous humor (10) which is influenced by several physical factors. The goal of the prevention of progression and the support of treatment in the case of developed high tension glaucoma is maintain the IOP in lower and steady values. Also, quick IOP changes should be preceded.

Increased intracerebral pressure seems to be one of the main causes of all high-altitude problems (11). Several studies have demonstrated the significant effect of altitude on IOP, whereas the investigated results are still controversial (3, 4, 6, 12–15). The effect of terrestrial altitude on IOP may be masked by a number of factors such as short-time physical activity (16) or changes in temperature (17, 18) etc., all in dependence on the level of fitness (16, 19, 20) and diurnal variations (21, 22). Strenuous exercise and weightlifting are associated with IOP rises (23, 24). In contrast, moderate aerobic physical activity causes its decrease (16, 25, 26). Maximal aerobic activity leads to high variability of inter-individual response of IOP (27).

The altitude changes result in two important effects–changes in air pressure and consequently changes in the partial pressure of the inspired oxygen. Recent studies have mostly evaluated both factors together in the form of hypobaric hypoxia, i.e., hypoxia induced by a decrease in the breathed ambient air pressure due to increase in altitude. These conditions were simulated in a hypobaric chamber (4, 12, 15) or achieved during climbing at a terrestrial high altitude (3, 6, 14, 15, 28). The authors of these studies published various results–an increase in IOP (3, 4, 6, 12) as well as decreasing (13–15). The observed changes in IOP were mostly evaluated as clinically insignificant, i.e.,> −2 mmHg and < 2 mmHg (29). Whether the IOP decrease or increase, a sufficiently long stay (days) at a high altitude (3, 6, 14, 15) leads to its stabilization and return to baseline, which can be a symptom of acclimatization. Generally, body acclimatization to a low oxygen content air mainly depends on genetics properties (30).

Thus, the effect of hypoxia on the IOP is not clear and the respective mechanism is not known. As all the above-mentioned studies evaluated both individual components of altitude change on IOP (changes in partial oxygen pressure and atmospheric pressure) together, there is a need to explain separately the contribution of each component. The main purpose of this study was to assess the IOP response to normobaric hypoxia (fraction of inspired oxygen FiO_2_ = 9.6%, simulated altitude ~6,200 m), i.e., without the effect of atmospheric pressure decrease, and the consequent recovery under other well-controlled ambient conditions. We also hypothesized, that this response will be independent on resting arterial oxygen saturation SpO_2_ and sex. As previous studies shown (16, 21), the IOP is correlated with individual basal heart rate (HR) as an indicator of fitness level. This variable was involved as the observed parameter during the experiment. The blood pressure was not observed due to insignificant relationship between its acute changes at altitude (and induced hypoxic changes) and changes in IOP (6). Moreover, the insignificant correlation of short-time changes of blood pressure and IOP was also found in some previous studies focused on moderate exercise (31, 32) except isometric exercise (23).

## Materials and Methods

### Participants

Forty-nine participants were originally recruited for the study. Only 38 visually healthy volunteers (23 women and 15 men) with an average age 25.2 ± 3.8 years met the inclusion criteria and performed the complete test (see below). The subjects were not allowed to have any evidence of either glaucomatous optic neuropathy or ocular hypertension. The subjects were also required to be free of ocular diseases which could affect IOP or its measurement such as keratoconus or high corneal astigmatism (equal or >-2.50 dioptre). All subjects were also free of cardiovascular, pulmonary and metabolic conditions and had not been exposed to hypoxia above 1,000 m for at least the previous 2 years.

This study was carried out in accordance with the recommendations of the ethics committee of the Faculty of Physical Culture, Palacký University Olomouc, Czech Republic. The protocol was approved by the ethics committee of the Faculty of Physical Culture, Palacký University Olomouc, Czech Republic, reference number 17/2016. All subjects gave written informed consent in accordance with the Declaration of Helsinky.

### Experimental Procedures

**Figure 1:**
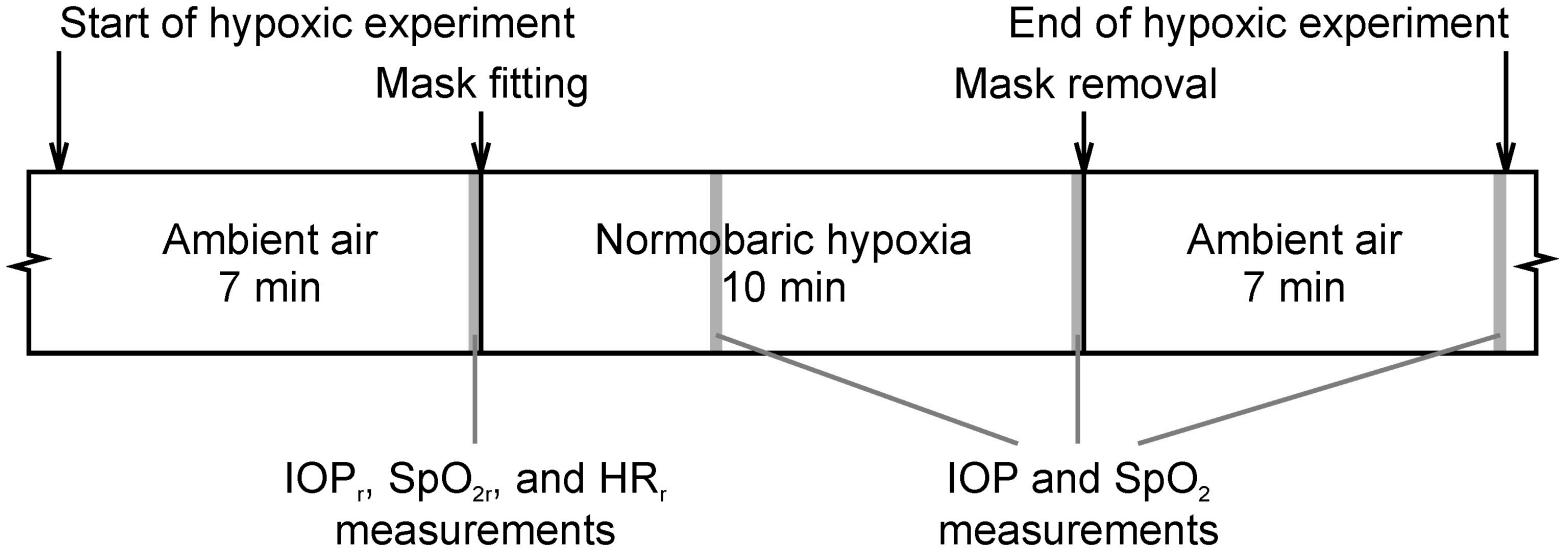
Course of the hypoxic experimental protocol. Gray colored areas depict the 10 s windows intended for measurement of intraocular pressure (IOP), arterial oxygen saturation (SpO_2_), and heart rate (HR).

The subjects were in a sitting position during the entire experiment. The initial resting IOP (*IOP*_r_), resting heart rate HR (*HR*_r_), which approximately indicates physical fitness level, and resting arterial oxygen saturation SpO_2_ were measured after 7 min of rest and were established as the baselines. The subjects breathed air corresponding to an altitude 250 m above sea level (established as normal conditions) during rest. Each subject then underwent the 10 min normobaric hypoxic period of the experiment and a subsequent 7 min recovery (see Figure 1). IOP and SpO_2_ were simultaneously re-measured at 4 and 10 min during the hypoxic period and at the end of the recovery period (i.e., at 7 min after hypoxia termination). Short-term hypoxia was chosen to limit the possible corresponding risks. During the hypoxic period, the subject breathed the gaseous mixture with the reduced partial oxygen pressure corresponding to the altitude 6,200 m above sea level, but under normal pressure (i.e., equal to the pressure at 250 m above sea level). This simulated altitude condition was created by using a MAG-10 system (Higher Peak, Boston, MA, USA), which simulated the lower O_2_ pressure found at high altitudes by lowering the percentage of O_2_ in the air at a similar atmospheric pressure (normobaric hypoxia). Subjects breathed air with a reduced O_2_ concentration via a face mask from a non-rebreathing circuit with a bag acting as a reservoir. The normobaric hypoxia condition, equal to the altitude of 6,200 m (FiO_2_ = 9.6 %), has been widely used in literature for intermittent hypoxic exposure (33). During the recovery period, the subject breathed ambient air once again. All the measurements were taken in the morning, this being the optimal time to eliminate the effect of circadian oscillations of IOP. During all the periods of experiment, the ambient temperature was maintained between 22 and 24°C, and the relative humidity was between 40 and 60%. Ten subjects out of the original number 49 were eliminated from the experiment because they were unable to withstand the 10 min hypoxia exposure and withdrawn prematurely; another one was eliminated due to technical failure of the measuring equipment.

### IOP, HR, and SpO_2_ Measurement

IOP was always measured in the sitting position using Icare Pro^®;^ rebound tonometer (Vantaa, Finland; www.icaretonometer.com). The tonometer averaged six automatically measured consecutive readings and provides their mean IOP out, which was used in the analysis. The coefficient of variation of the output (the automatically averaged IOP value) declared by the manufacturer is <8% in accordance with publications (34, 35). Only the right eye of each participant was measured. All IOP measurements were administrated by one trained professional.

The SpO_2_ was continuously measured using a Nonin Avant 2120 pulse oximeter (Nonin Medical, Minneapolis, MN, USA; http://www.nonin.com) set on the right index finger. The SpO_2_ was measured at a sampling frequency of 1.0 Hz, and the average of 10 readings was calculated for the subsequent statistical analysis. The accuracy of the output expressed as standard deviation declared by the manufacturer is 2%.

To determine the HR, the ECG was recorded at a sampling frequency of 1,000 Hz using DiANS PF8 diagnostic system (Dimea Group, Olomouc, Czech Republic). The system includes a chest strap, unit for recording and transmitting ECG data, and a receiver connected to a personal computer with special software. HR was calculated from the ECG record of a duration of 10 s.

### Statistical Analysis

The measured IOP and SpO_2_ data and influence of sex were analyzed by two-factor repeated-measures ANOVA (time as a within-participants factor, sex as a between-participants factor). The dependence of IOP changes on *HR*_r_, *IOP*_r_ or changes of SpO_2_ were studied with the Pearson correlation coefficient (*r*). The significance level was set at 0.05. When necessary, the levels of statistical significance included a Huynh-Feldt correction for departures from sphericity. The *post-hoc* pairwise comparisons were realized using Tukey honest significant difference (HSD) test; the Cohen's *d* as a measure of effect size is reported as well. Data are presented as mean ± standard deviations. Statistical analyses were performed using STATISTICA 13.0 (StatSoft, Tulsa, OK, USA).

## Results

**Figure 2:**
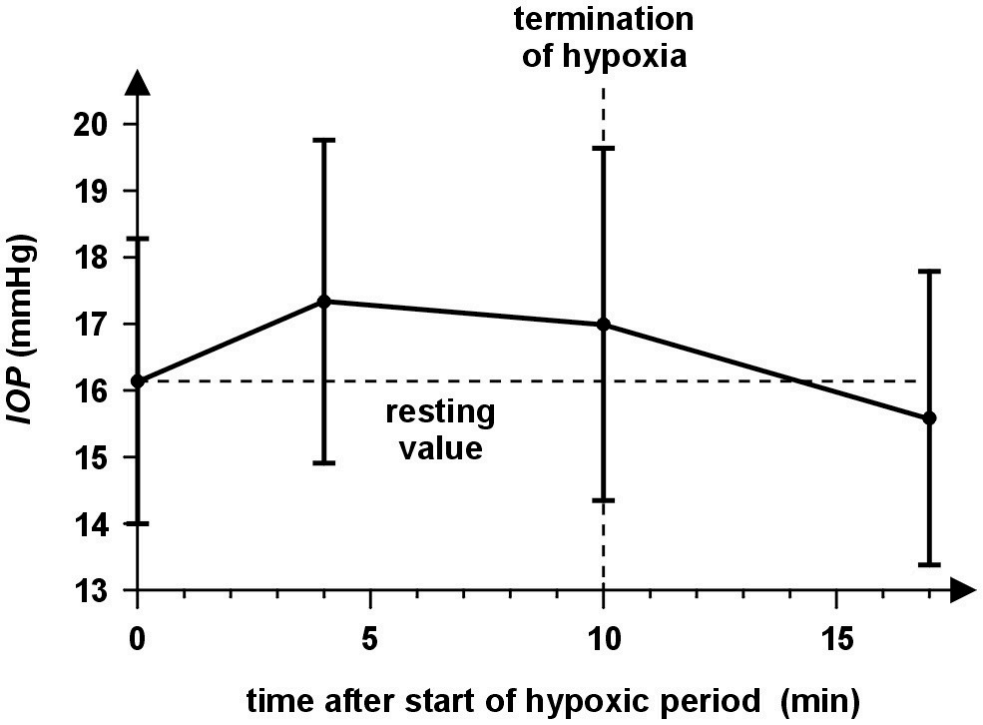
The time course of IOP during the experiment for all subjects. The circles represent the mean IOP values of particular measurement before the hypoxic period (0 min), at minute 4 and 10 during 10 min normobaric hypoxia and at the end of the 7 min recovery (17 min). The half-sizes of the vertical abscissae correspond to the IOP standard deviations. IOP increases during both measurements in the hypoxic period and returned approximately to the resting value at 7 min of recovery if averaged across all subjects.

The mean values and standard deviations of the IOP during the experiment are presented in Figure 2. The ANOVA revealed that the values of IOP altered significantly [*F*_(3, 108)_ = 12.16, *p* < 0.001] with time. A statistically significant increase in IOP compared with the baseline (*IOP*_r_ = 16.0 mmHg ± 2.2 mmHg) was observed during the hypoxic period with the mean difference 1.2 mmHg ± 1.9 mmHg at minute 4 and 0.9 mmHg ± 2.3 mmHg at minute 10 (*post-hoc* Tukey HSD test, *p* < 0.001, *d* = 0.642 and *p* = 0.027, *d* = 0.366); both hypoxic IOP values did not differ significantly from one another (*p* = 0.68, *d* = 0.210). The maximal observed individual IOP increases were 5.3 mmHg and 6.2 mmHg in minutes 4 and 10 (for different subjects), respectively, and 32 and 34% values increased more than 2 mmHg. The IOP returned to the baseline at 7 min recovery (*post-hoc* Tukey HSD test, *p* = 0.27, *d* = 0.333). The main effects of sex was insignificant [*F*_(1, 36)_ = 0.22, *p* = 0.64] as well as its interaction with time [*F*_(3, 108)_ = 2.28, *p* = 0.084].

We also studied the effects of the *HR*_r_ and the baseline *IOP*_r_ on differences Δ*IOP* from the baseline (hypoxic value minus baseline). The correlation analysis had shown insignificant correlations between *IOP*_r_ and Δ*IOP* at minute 4 (*r* = −0.273, *p* = 0.097) and at minute 10 (*r* = −0.311, *p* = 0.058) of the hypoxic exposure. Correlations between *HR*_r_ and Δ*IOP* were also insignificant (*r* = −0.106, *p* = 0.53 and *r* = −0.014, *p* = 0.94) at minutes 4 and 10, respectively.

**Figure 3:**
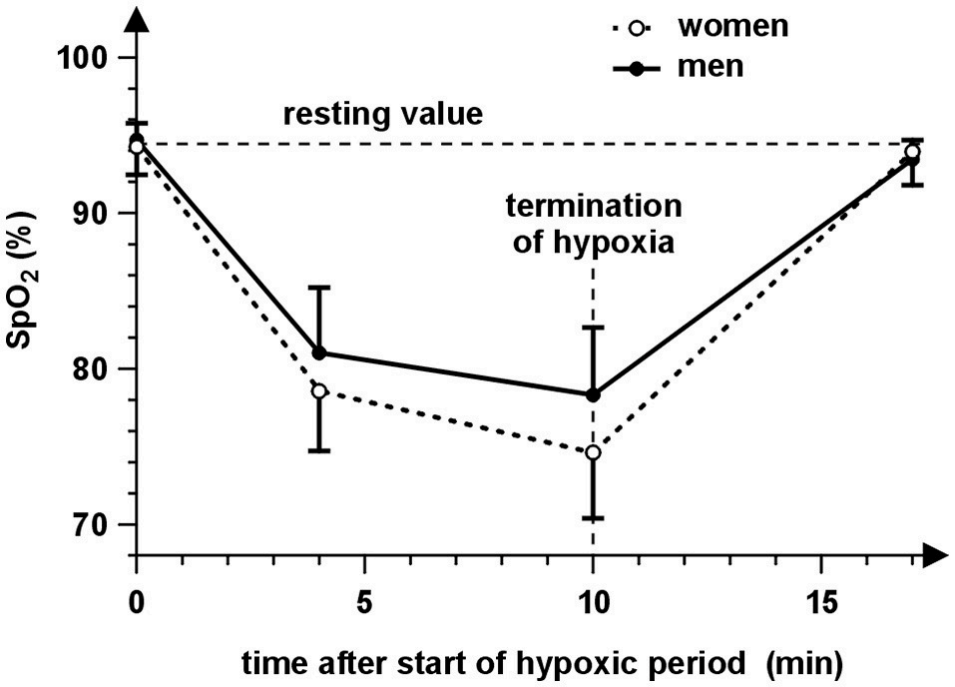
The time course of arterial oxygen saturation (SpO_2_) during the experiment for women (open symbols) and men (close symbols). The circles represent the mean SpO_2_ values of the particular measurement before the hypoxic period (0 min), at minute 4 and 10 during 10 min normobaric hypoxia and at the end of the 7 min recovery (17 min). The vertical abscissae correspond to the SpO_2_ standard deviations. It is evident that SpO_2_ decreases during hypoxia and returns to the resting value at 7 min of recovery. The values of SpO_2_ are lower during hypoxia in the case of women compared to men.

The SpO_2_ changed significantly with time [repeated-measures ANOVA with Huynh-Feldt correction, *F*_(1.8, 65.2)_ = 313.50, *p* < 0.001]. It decreased during the hypoxic period compared to the baseline (94.4% ± 1.6%) with the mean difference 14.9 ± 4.2% at minute 4 and 18.4 ± 5.8% at minute 10 and returned to the baseline at minute 7 after the hypoxic period end (*post-hoc* Tukey HSD test, *p* < 0.001, *d* = 3.595, *p* < 0.001, *d* = 3.173 and *p* = 0.79, *d* = 0.440, respectively). A decline in SpO_2_ was significantly higher in women compared to men [Figure 3; significant interaction of sex and time, *F*_(3, 108)_ = 3.34, *p* = 0.022]. The main effect of sex was insignificant [*F*_(1, 36)_ = 3.92, *p* = 0.055].

**Figure 4:**
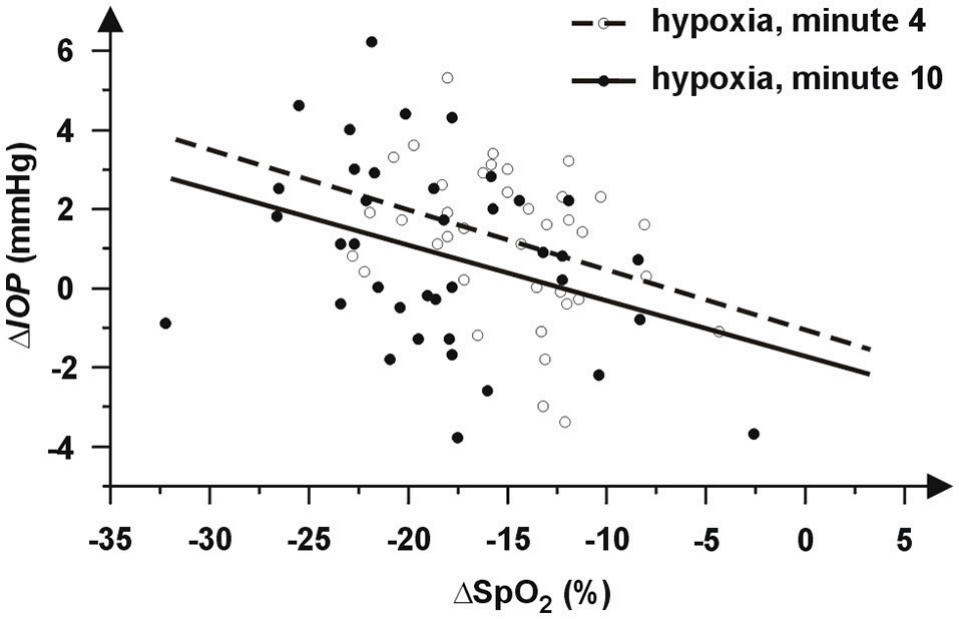
Graphic dependence of individual differences Δ*IOP* from its baseline *IOP*_r_ on changes of peripheral capillary oxygen saturation (ΔSpO_2_) at minute 4 (open circles) and minute 10 (closed circles). The negative values represent a decrease. The dependences are approximated by regression lines (dashed line at minute 4 and solid line at minute 10).

The comparison of graphs in Figures 2, 3 indicates a relationship between behavior of SpO_2_ and *IOP* during hypoxia. This is supported by significant correlations between changes of IOP and SpO_2_ at minute 4 (*r* = −0.337, *p* = 0.038) as well as minute 10 (*r* = −0.346, *p* = 0.033). The corresponding dependences are presented in Figure 4.

## Discussion

This study was focused on determining IOP response to short-term normobaric hypoxia (FiO_2_ = 9.6%, simulated altitude ~ 6,200 m). The primary finding of this research was a statistically significant increase in IOP as an immediate response to hypoxia (see Figure 2). Our findings are in accordance with some previously published studies, which reported the IOP increase at various altitudes (9,144 m and 5,490–7,625 m above sea level) simulated in a hypobaric chamber (4, 12, 13) as well as in the case of actual climbing up to 5,200 and 6,265 m above sea level (3, 6). The reported mean IOP increases were below 2 mmHg expect observation of Ersanli et al. (12), which published an increase in IOP from the base line 12.31 ± 2.98 mmHg up to 14.37 ± 3.44 mmHg during hypoxia simulated in the hypobaric chamber. The average IOP value did not differ from the baseline in 30 min after leaving the chamber. Other studies, in contrast, showed a decrease in IOP when hiking at a terrestrial altitude [5,050 m above sea level (14) and 4,300 m above sea level (15)] and also in a hypobaric chamber (15). These studies were performed in different ways to reach a different altitude level and method of own measurement which can be responsible for these differences. The studies including our study mostly used hand-held tonometers-tono-pen (3, 4, 6, 12, 14, 15) or Icare (13). The disadvantage of the hand-held tonometers is lower accuracy in comparison with the Goldmann applanation tonometer. The coefficients of variation of these tonometers are about 8% (34–37). With regard to the mean IOP, the standard deviation of measurement can be considered about 1.3 mmHg, which is close to observed changes and can affect significance of measured data. In all the presented studies, apart from our research, each subject was exhibited simultaneously to the hypoxia and reduced atmospheric pressure. Moreover, in the case of actual hiking, the climb to the given altitude was connected with the physical aerobic activity, which results in IOP decrease (16), and/or to changes of another physical parameter of the surrounding environment. Thus, the subjects were affected by many different parameters, whose joint effect may result in different IOP responses. In our study, the hypoxia was reached by a sudden change of partial oxygen pressure without the possibility of adaptation, which affects significantly IOP changes (3, 6, 14, 15). Other important factors, especially atmospheric pressure, ambient temperature and humidity were unchanged.

It is known that the corneal thickness increases (due to edema) at a higher altitude (3, 13, 38) and thicker corneas reveal an improperly higher IOP reading (39). McNamara (40) and Wang (41) consequently determined that 1 h and longer corneal hypoxia causes an increase in the corneal thickness. These effects together could explain the IOP increase at higher altitudes or during hypoxia. Karadaq et al. (4) and Somner at al. (6) considered the corneal thickness correction of IOP readings. Their results still revealed, however, an IOP increase at a high altitude. With regard to the short time of hypoxia in our experiment, we considered that its effect on the thickness was minimal if any. Moreover, the discussed short-time corneal changes could not cause wide changes of anatomy and relevant biomechanical corneal properties, thus their effect on the IOP reading should be minimal in our experiment. We therefore suppose that the observed IOP increase was not linked to the corneal thickening.

Our results also revealed a correlation between IOP changes and changes in blood oxygen saturation (see Figure 3). The higher decrease in oxygen saturation is related to the higher IOP increase. The SpO_2_ decrease was consistent with previous findings (42). Moreover, a higher degree in hypoxia (lower values of SpO_2_) was reached with women. We could therefore assume a stronger effect of hypoxia in the case of women. Nevertheless, the relationship between sex and IOP was evaluated as statistically insignificant as well as the interaction of sex and time. Our data also did not show relationship between IOP changes and IOP baseline or initial HR. However, presented results can be negatively influenced by the variability of the IOP measurement, which can mask true effects and can decrease the statistical power of used tests, especially in the case of analysis of IOP changes. If the average standard deviation of the IOP measurement is about 1.3 mmHg, the standard deviation of IOP changes (computed using common error propagation rule) is about 1.3 × √2 mmHg = 1.8 mmHg. Thus, the effects of sex and IOP and HR baselines need verification in a future study with a larger sample size.

The short-term exposition of the normobaric hypoxia causes a small mean increase of IOP, which seems to be marginal (below 2 mmHg), and from a medical standpoint clinically irrelevant (29). More than 30 % of individual IOP values measured during hypoxia exceed this limit, however, and the maximal change was about 6 mmHg. Such a quick change yields risk for these people, especially in the case of presence glaucoma (9). Moreover, glaucoma patients are more sensitive to stress test, e.g., water-drinking test (43), than healthy subjects. Thus, the IOP changes induced by hypoxia in glaucoma patients may be greater that those we found. On the other hand, the change is only short-term. We also determined that the IOP rising increases with decreasing SpO_2_. Thus, we recommended monitoring of IOP during activities connected with short-term high hypoxia (such as intermittent hypoxic training (44) or with quick changes in altitude) in the case of people with glaucoma, glaucoma suspected or people with a higher risk of glaucoma (such as people with diabetes etc.).

## Conclusion

We found an increase in IOP in response to short-term normobaric hypoxia, which returned to the baseline 7 min after hypoxia. The increase was higher for subjects with a higher degree of induced oxygen desaturation. Although the average increase was clinically insignificant, clinicians should be aware that some patients who perform the activities connected with short-term hypoxia may run the risk of an unsafe increase in intraocular pressure.

## Data Availability

The raw data supporting the conclusions of this manuscript will be made available by the authors, without undue reservation, to any qualified researcher.

## Author Contributions

EN and MB Study design. EN, JK, and JJ Data collection. FP and JK Data analyses. All authors contributed to the data interpretation, drafting the manuscript and manuscript revision, read and approved the submitted version.

### Conflict of Interest Statement

The authors declare that the research was conducted in the absence of any commercial or financial relationships that could be construed as a potential conflict of interest.
